# Effectiveness of islatravir post‐exposure prophylaxis after intravenous challenge with simian immunodeficiency virus in rhesus macaques

**DOI:** 10.1002/jia2.26507

**Published:** 2025-06-18

**Authors:** Martin Markowitz, Agegnehu Gettie, Leslie St. Bernard, Brooke Grasperge, Ryan Vargo, Michelle Pham, Kerry Fillgrove, Neal Dube, Tracy L. Diamond, Daria J. Hazuda, Jay A. Grobler

**Affiliations:** ^1^ Aaron Diamond AIDS Research Center New York New York USA; ^2^ Tulane National Primate Research Center Covington Louisiana USA; ^3^ Merck & Co., Inc. Rahway New Jersey USA

**Keywords:** islatravir, post‐exposure prophylaxis, rhesus macaque, SIV, pharmacokinetic, islatravir‐TP

## Abstract

**Introduction:**

Islatravir (ISL) is a nucleoside reverse transcriptase translocation inhibitor (NRTTI) with robust antiretroviral activity. The efficacy of ISL administered for post‐exposure prophylaxis (PEP) was evaluated in a simian immunodeficiency virus (SIV) rhesus macaque intravenous (IV) challenge model.

**Methods:**

Twelve rhesus macaques were challenged with SIVmac251 via IV administration. After 24 hours, six animals received ISL 3.9 mg/kg (the minimum effective dose that gives maximal protection) and six animals were untreated controls. In stage 1, treated animals received 4 weekly oral doses of ISL and were monitored for SIV infection for 7 weeks after the last dose. In stage 2, uninfected, treated animals from stage 1 were challenged similarly; 24 hours after challenge, 3 weekly oral doses of ISL 3.9 mg/kg were initiated. The treated animals were monitored for 7 weeks, as in stage 1. Uninfected, treated animals (from stage 2) entered stage 3. In stage 3, the animals were challenged as in stage 2; 24 hours after challenge, 2 weekly oral doses of ISL 3.9 mg/kg were initiated. The treated animals were monitored for 7 weeks, as before. Finally, in stage 4, uninfected, treated animals were challenged using IV administration and 24 hours later were treated with a single oral dose of ISL 3.9 mg/kg and monitored for 7 weeks. Infection was monitored through plasma viral RNA and proviral DNA amplification. Virus‐specific antibody responses were measured using a commercial assay. ISL concentrations in plasma and ISL triphosphate (ISL‐TP) levels in peripheral blood mononuclear cells were measured longitudinally.

**Results:**

All untreated controls were viraemic 7 days after SIVmac251 IV challenge. All six ISL‐treated animals were completely protected in stages 1–3 (Fisher exact test *p* = 0.0022). In stage 4, two of six ISL‐treated animals became infected with wild‐type SIVmac251: viraemia was observed at days 14 and 49 in the two animals (Fisher exact test *p* = 0.06). Both animals had unquantifiable ISL‐TP on the day viraemia was observed.

**Conclusions:**

Two weekly oral doses of ISL 3.9 mg/kg, administered 24 hours post IV SIV exposure, prevents infection of rhesus macaques. These results support further investigation of a long‐acting oral NRTTI for PEP.

## 1 INTRODUCTION

Evidence regarding the effectiveness of post‐exposure prophylaxis (PEP) in the prevention of HIV acquisition in humans comes from one case‐control study; studies using animal models further support its use [1−3]. The standard of care for PEP against HIV‐1 acquisition is a combination of antiretroviral agents taken daily for 28 days after exposure [4−6]. The recommended PEP regimens vary slightly by country or region but typically comprise a two‐ or three‐drug regimen, often including an integrase strand transfer inhibitor [4−6]. A 28‐day duration is standard, as is the guidance to initiate PEP ideally within 24 hours and no later than 72 hours after potential exposure to HIV [4−6].

Islatravir (ISL) is a nucleoside reverse transcriptase translocation inhibitor (NRTTI) with activity against wild‐type HIV‐1 and variants with common nucleoside reverse transcriptase inhibitor resistance‐associated substitutions. It exhibits no known cross‐resistance to other classes of antiretroviral agents [7−15]. Its sub‐nanomolar potency, resistance profile, tolerability, long half‐life and broad tissue distribution make it ideal for daily and weekly dosing for HIV‐1 treatment [16, 17]. ISL is rapidly converted to its active form, ISL triphosphate (ISL‐TP), within target cells where it inhibits HIV‐1 reverse transcriptase activity.

The robust antiviral activity of ISL observed in vitro has translated to preclinical in vivo studies and the clinic. Activity of ISL against HIV‐1, HIV‐2 and simian immunodeficiency virus (SIV) has been demonstrated in cell lines and human or rhesus monkey primary cells [18, 19]. As anticipated based on the potency and pharmacokinetics (PK) of ISL and ISL‐TP, once‐weekly oral doses of as low as 3.9 mg/kg reduced mean viral load by >1.0 log_10_ copies/ml for 1 week in SIV‐infected rhesus macaques [20]. In a previous pre‐exposure prophylaxis (PrEP) study in macaques, once‐weekly oral doses of ISL 0.1−3.9 mg/kg also protected macaques against repeated rectal exposure to simian/human immunodeficiency virus (SHIV) [21].

In treatment‐naive adults living with HIV‐1, single oral doses of ISL (0.5−30 mg) significantly suppressed HIV‐1 RNA by >1.0 log by day 7 [17]. The combination of oral ISL 0.25 mg and doravirine 100 mg once daily is being evaluated in Phase 3 studies for the treatment of adult participants living with HIV‐1 who are switching their antiretroviral regimens, and also in treatment‐naive individuals living with HIV‐1 [22, 23]. The combination of ISL 2 mg and lenacapavir 300 mg once weekly is being evaluated as a treatment for participants living with HIV‐1 in Phase 3 studies [24, 25]. The objective of the current study was to investigate if weekly doses of ISL administered as PEP can provide protection against SIV in a macaque model [26].

## 2 METHODS

### 2.1 Animals

Twelve Indian rhesus macaques—six that were administered ISL and six that did not receive PEP—were used in the challenge study. The study was approved by the Institutional Animal Care and Use Committee of Tulane National Primate Research Center (TNPRC). The accreditation of TNPRC is provided by the Association for Assessment and Accreditation of Laboratory Animal Care (AAALAC #000594). TNPRC's OLAW animal welfare assurance number is A4499‐01 and USDA registration is 72‐R‐0002.

### 2.2 SIVmac251 viral stock

SIVmac251 viral stock, produced in macaques, was supplied by Dr Ron Desrosiers. The titre of the stock was 5×10^3^ the 50% tissue culture infectious dose per millilitre on CEMx174 cells using the methods described by Reed and Muench [27].

### 2.3 SIVmac251/rhesus macaque challenge

**Figure 1:**
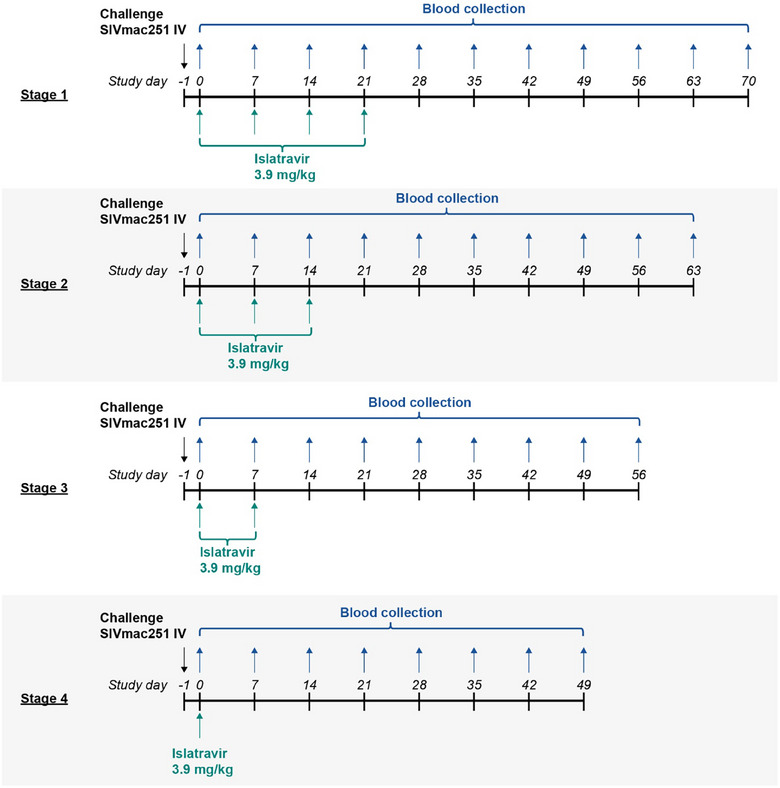
Rhesus macaque SIVmac251 challenge model with PEP dosing at different stages. The black arrow shows the schedule of SIVmac251 inoculations at day −1. The blue arrows show the schedule of blood collection, and the green arrows show the schedule of weekly doses by oral gavage of ISL 3.9 mg/kg. Uninfected animals in stages 1–4 were then challenged as in stage 1 for each subsequent stage: receiving 3, 2 and 1 weekly dose of oral ISL, respectively, initiated 24 hours after inoculation and monitored for 7 weeks after the last dose. ISL, islatravir; PEP, post‐exposure prophylaxis; SIVmac251, simian immunodeficiency virus mac251.

In stages 1–4, 12 macaques were challenged at day −1 by intravenous (IV) administration of 10 times the 50% animal infectious dose (10×AID_50_) of SIVmac251. After 24 hours (day 0), six animals received 4 (stage 1), 3 (stage 2), 2 (stage 3) or 1 (stage 4) weekly doses of ISL 3.9 mg/kg in 10% Tween 20 vehicle by oral gavage, and six received 4 weekly doses of vehicle only (untreated controls). In stages 2−4, uninfected animals from the treated group in the previous stage were used and challenged prior to dosing as in stage 1. Across all stages, animals were monitored for SIV infection for 7 weeks after the last dose (Figure 1).

### 2.4 Virologic and immunologic assessments

Animals were monitored for signs of infection using a reverse transcription polymerase chain reaction viral load assay from plasma samples with a lower limit of detection (LLD) of 40 SIV RNA copies/ml and peripheral blood mononuclear cells (PBMCs) proviral DNA amplification (200–500 ng/sample; five determinations per time point). Sequencing of reverse transcriptase from plasma RNA was performed for animals that acquired SIV infection after prophylaxis with ISL. Virus‐specific antibody responses were measured by enzyme‐linked immunosorbent assay (ELISA) using commercial SIVmac251 gp120‐coated plates (ImmuneTech IT‐001‐156p). Plasma ISL and ISL‐TP concentrations in PBMCs were measured longitudinally in each stage (for 7 weeks after the last dose).

### 2.5 PK assessments

The concentrations of ISL in plasma and ISL‐TP in PBMCs were determined by liquid chromatography‐tandem mass spectrometry 7 days after dosing (trough concentration), as described previously [21]. ISL‐TP trough concentration has previously been correlated to efficacy levels obtained in the clinic [16].

To compare observed ISL‐TP trough concentrations in animals with concentrations observed in the clinic, a population PK model was used based on plasma ISL and PBMC ISL‐TP concentrations from Phase 1−3 studies. Simulations were performed in a typical population (*n* = 1000) with inter‐individual variability on PK parameters.

### 2.6 Statistical analysis

The log‐rank test was used to calculate statistical differences between the ISL‐treated animals in each stage compared with the vehicle‐treated control animals. Hazard ratios were estimated using the log‐rank model. All analyses were performed using GraphPad Prism, v7.0.

## 3 RESULTS

### 3.1 Effectiveness of ISL as PEP

**Figure 2:**
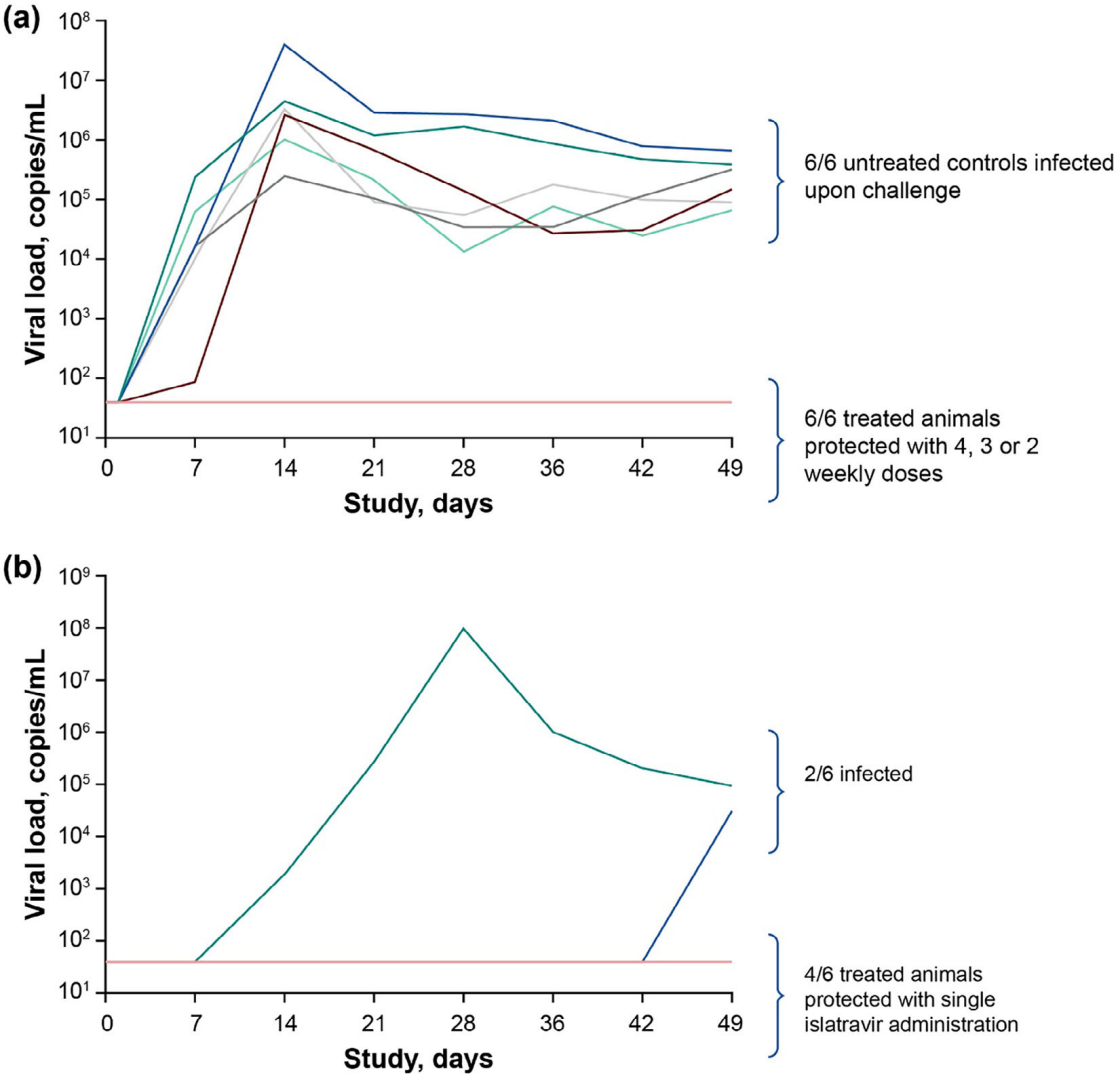
(a) Plasma viral load through day 49 in rhesus macaques for untreated control animals in stage 1 and treated animals in stages 1–3 after SIVmac251 IV inoculation, followed by 2−4 weekly doses of ISL 3.9 mg/kg by oral gavage. Undetectable viral loads are displayed at the LLD (40 copies/ml). The coloured lines represent individual animals. (b) Plasma viral load through day 49 in infected rhesus macaques in stage 4 after SIVmac251 IV inoculation followed by a single dose of ISL 3.9 mg/kg by oral gavage. The coloured lines represent individual animals. ISL, islatravir; IV, intravenous; LLD, lower limit of detection; SIVmac251, simian immunodeficiency virus mac251.

Control animals were challenged by IV administration of 10×AID_50_ SIVmac251 in the absence of ISL PEP; all control animals had detectable viraemia 7 days after challenge. Six animals in the PEP arm received 1–4 weekly doses of ISL initiated 24 hours after challenge with IV administration of SIVmac251 depending on the stage (Figure 1). The macaques that were treated with 4, 3 or 2 weekly doses of ISL (in stages 1−3, respectively) as PEP starting 24 hours after SIVmac251 challenge were completely protected (no observed viraemia above the LLD for at least 7 weeks after the last dose of ISL; Figure 2 and data not shown). Non‐infection was confirmed by the absence of both proviral DNA and antibodies to SIV by ELISA. In stage 4, animals were given one dose of ISL within 24 hours of the viral challenge. Two of six animals in stage 4 became infected with SIVmac251; viraemia was detected at day 14 in one animal and at day 49 in the other (Figure 2). Genotyping confirmed that both animals were infected with a virus encoding wild‐type reverse transcriptase. The data indicate ≥39.12‐fold (*p* = 0.0009, by log‐rank test) lower risk of infection than in control animals at each stage.

### 3.2 Intracellular ISL‐TP

**Figure 3:**
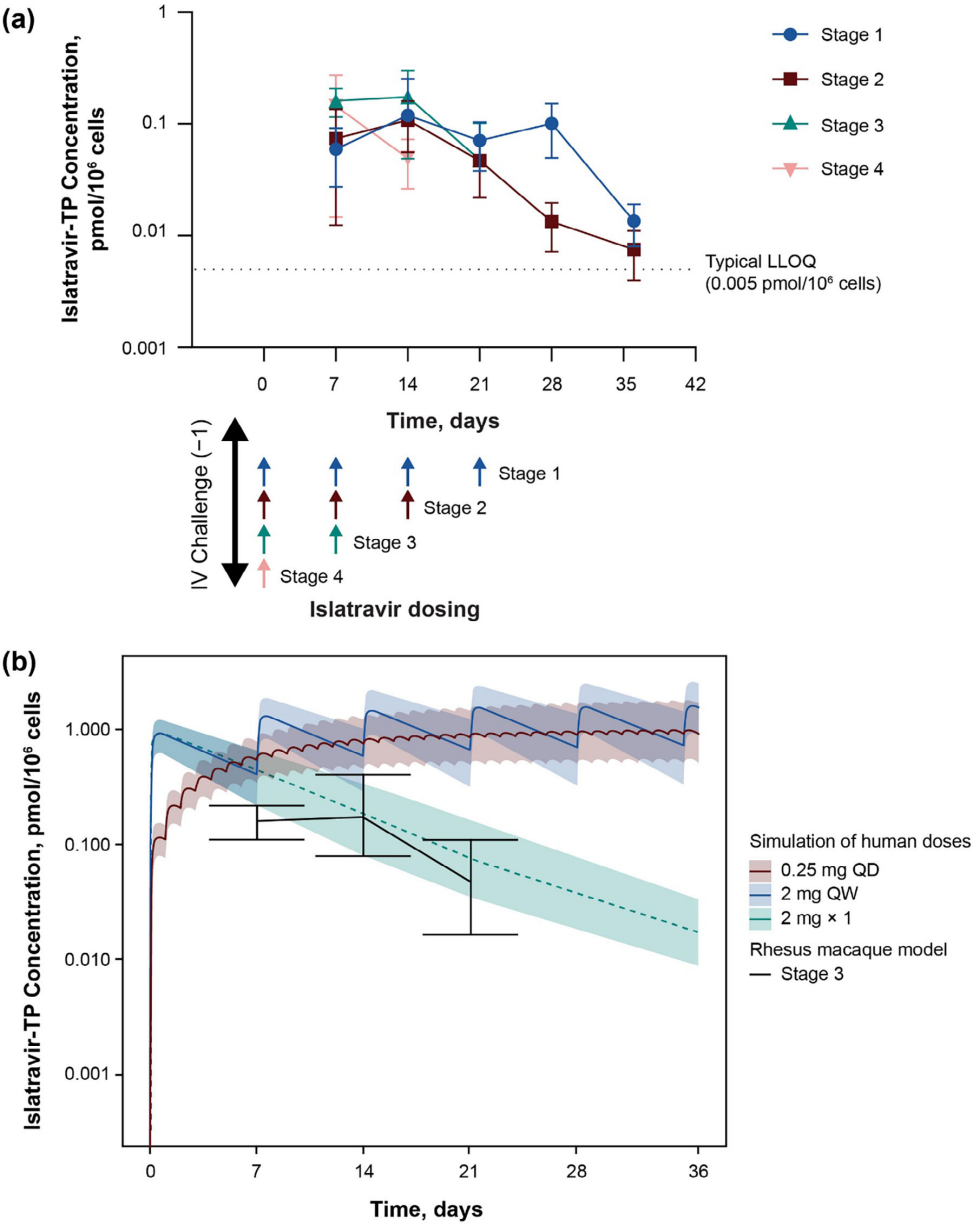
(a) Mean weekly intracellular ISL‐TP concentration levels starting on day 7 in all four stages after 1−4 weekly oral doses of ISL 3.9 mg/kg. The arrows represent the timing of ISL dosing per stage. The figure includes data only if there were at least two measurements with quantifiable ISL‐TP at each specific time point for each stage. ISL‐TP was below the LLOQ in rhesus PBMCs from all animals 3 weeks after the last dose of ISL 3.9 mg/kg. (b) Comparison of rhesus ISL‐TP exposure to simulated exposure of ISL‐TP in humans after once‐daily oral dosing of ISL 0.25 mg, once‐weekly oral dosing of ISL 2 mg or a single dose of ISL 2 mg. Coloured lines and bands represent the simulated median and 95% prediction interval, respectively, for dosing in humans. The solid black line represents the observed mean concentrations with minimum and maximum concentrations represented in the error bars in stage 3 in macaques. ISL, islatravir; ISL‐TP, ISL‐triphosphate; IV, intravenous; LLOQ, lower limit of quantitation; PBMC, peripheral blood mononuclear cell; QD, once daily; QW, once weekly.

The ISL‐TP levels 7 days after each ISL dose were determined in PBMCs isolated from macaques in stages 1–4. There was a range of ISL‐TP from 0.005 to 0.407 pmol/10^6 ^cells for quantifiable values across all animals and stages (Figure 3a). Because stage 3 (2 weekly doses of ISL) was the stage with the fewest ISL doses providing complete protection, the ISL‐TP exposures seen in macaques in stage 3 were compared with those simulated for the two ISL dose levels currently in clinical development for HIV‐1 treatment (ISL 0.25 mg once daily and ISL 2 mg once weekly). As shown in Figure 3b, the levels simulated for the two human doses are above the protective levels observed in macaques, and the level for a single 2‐mg dose is similar to the levels observed in macaques.

## 4 DISCUSSION

Previous PEP studies in macaques showed that the timing of initiation and duration of treatment were critical to prevent infection [28−30]. Macaques that received prophylactic tenofovir (TFV) subcutaneously once daily 24 hours after exposure and continued for 4 weeks were protected from SIV_mne_ infection [31]. In another study, viral replication was inhibited in macaques that received TFV 1 week after exposure with SHIV_KU2_ [32]. However, even with viral loads below the LLD during 12 weeks of daily TFV, the virus was not cleared, emphasizing the importance of early prophylaxis [32]. Results of two other studies in macaques suggest that the first week after SIV/SHIV exposure in macaques (and perhaps HIV‐1 exposure in humans) is a critical window for the effectiveness of PEP [2, 3], underscoring the imperative that any PEP regimen should be easily and quickly available to be given after HIV‐1 exposure to prevent acquisition.

To test whether weekly ISL is effective as PEP in a macaque challenge model, we selected the minimum dose (3.9 mg/kg) that had maximal efficacy in a previous SIVmac251 treatment study [20]. Animals were challenged with IV administration of SIVmac251, and the number of weekly 3.9 mg/kg doses varied between stages. In the current study, we demonstrate that 2 weekly oral doses of ISL 3.9 mg/kg started 24 hours after SIVmac251 challenge prevented infection in macaques (stage 3). This complete protection correlates with ISL‐TP levels maintained at a mean trough concentration of 0.05 pmol/10^6^ cells through 21 days after treatment initiation. Although a single dose of ISL given 24 hours after SIVmac251 challenge (stage 4) statistically provided protection from infection, two of six animals in this stage acquired SIV. The ISL‐TP levels in stage 4 were only quantifiable through day 14. These data raise the question of whether a single post‐exposure dose might provide sufficient protection for humans.

Although 2 weekly doses were necessary for complete protection in the macaque model, the terminal half‐life of ISL‐TP in human PBMCs (∼190 hours) is substantially longer than the half‐life of ISL‐TP in macaque PBMCs (∼50 hours) [16, 17, 20, 33]. The longer half‐life in humans maintains higher concentrations of ISL‐TP for a longer period, consistent with the exposure in stage 1, 2 or 3 of this study. Therefore, a single oral dose of ISL given as PEP within 24 hours of HIV exposure could provide a protective effect.

Single‐dose oral PEP could be stocked in various settings for quick and easy access to address the need for urgent initiation of PEP, which is ideally within 24–48 and no later than 72 hours, to prevent HIV acquisition. Furthermore, a single dose of oral PEP may remove the adherence requirements of a 28‐day regimen. Lastly, PEP regimens must be well tolerated and have minimal drug−drug interactions to be appropriate for the diverse demographics who may need PEP, both of which are attributes of ISL and possibly of other compounds in the NRTTI class.

Although the clinical development of oral once‐monthly ISL for HIV PrEP was discontinued due to dose‐dependent decreases in total lymphocyte and CD4+ T‐cell counts seen across the ISL development programme [34, 35], clinical investigations, together with modelling data, identified an ISL‐TP threshold of 9 µM (∼1.8 pmol/10^6^ cells), below which decreases in lymphocytes are not expected [36]. ISL continues to be investigated for HIV‐1 treatment, and decreases in lymphocytes have not been observed at the ISL doses currently in development for treatment: 0.25 mg daily and 2 mg weekly.

MK‐8527 is a novel, structurally distinct NRTTI in development for monthly oral HIV‐1 prophylaxis [37, 38]. MK‐8527 was generally well tolerated in Phase 1 studies in people living with HIV and living without HIV‐1 [39, 40]. A Phase 2 study of monthly oral dosing of MK‐8527 (clinicaltrials.gov; NCT06045507) is ongoing, and Phase 3 studies of MK‐8527 for monthly oral PrEP are being planned. Although currently no compounds are formally indicated for use as PEP and adequately powered efficacy trials have inherent ethical and practical challenges, clinical data supporting the use of MK‐8527 for PrEP, combined with animal data demonstrating the efficacy of NRTTIs as PEP, may facilitate consideration of these novel compounds—which are potent, reach efficacious levels quickly, have a relatively long half‐life and extended dosing intervals—for PEP use.

## 5 CONCLUSIONS

Two weekly doses of ISL as PEP, administered 24 hours post IV SIV exposure, provided complete protection in the SIV/macaque model. Extrapolation to human PK suggests that a single oral dose of ISL could provide clinical protection. These results suggest a potential utility of a long‐acting oral NRTTI as simplified HIV‐1 PEP in humans.

## COMPETING INTERESTS

KF, ND, DJH, JAG, MP, RV and TLD are current or former employees of Merck Sharp & Dohme LLC, a subsidiary of Merck & Co., Inc., Rahway, NJ, USA, and may own stock and/or options in Merck & Co., Inc., Rahway, NJ, USA. DJH is an inventor on related patents. MM, AG, LSB and BG have no conflicts to report.

## AUTHORS’ CONTRIBUTIONS

All authors have read and approved the final manuscript. MM, KF, DJH and JAG substantially contributed to the conception and design of the study. RV, KF, ND, MP, TLD, BG, MM, AG, LSB and JAG worked on the acquisition, analysis or interpretation of data. RV, KF and TLD drafted the manuscript. TLD and MM critically revised the manuscript for important intellectual content.

## FUNDING

Funding for this research was provided by Merck Sharp & Dohme LLC, a subsidiary of Merck & Co., Inc., Rahway, NJ, USA.

## Data Availability

The data sharing policy, including restrictions, of Merck Sharp & Dohme LLC, a subsidiary of Merck & Co., Inc., Rahway, NJ, USA (MSD), is available at https://trialstransparency.msdclinicaltrials.com/policies‐perspectives.aspx. Requests for access to the study data can be submitted via email to the Data Access mailbox (mailto:dataaccess@msd.com).
